# Functional Outcomes in the Distal End of Radius Fracture: A Prospective Study in a Tertiary Care Center

**DOI:** 10.7759/cureus.74226

**Published:** 2024-11-22

**Authors:** Varun Thusoo, Arjun S Chakrapani, Ashish Nehru, Sachin Kudyar, Brahmpreet Nagpal, Alok KV, Ebin S, Akhil Jose

**Affiliations:** 1 Department of Orthopaedics, Royal Shrewsbury Hospital NHS Trust (SaTH), Shrewsbury, GBR; 2 Department of Orthopaedics and Traumatology, St. Thomas' Hospital, London, GBR; 3 Department of Orthopaedics, Employees State Insurance Corporation Hospital, New Delhi, IND; 4 Department of Orthopaedics, Government Medical Hospital, Jammu, IND; 5 Department of Orthopedics, Adesh Medical College and Hospital, Kurukshetra, IND; 6 Department of Orthopaedics and Traumatology, Osmania Medical College, Hyderabad, IND; 7 Department of Orthopedics and Traumatology, Kunhitharuvai Memorial Charitable Trust (KMCT) Medical College, Manassery, IND

**Keywords:** conservative treatment, distal radius fracture, functional outcomes, radiographic outcomes, surgical treatment

## Abstract

Objectives: The objectives of this study are to determine the functional outcomes and compare them between conservative and surgical management in patients managed for closed-type intra-articular distal end of radius fractures.

Methods: A prospective observational study was done on 150 patients who underwent treatment for closed-type intra-articular distal end of radius fractures. As per Frykman Classification, they were type III. Patients were either managed conservatively, i.e. 100 patients out of 150 and surgical management was done in 50 cases. For surgical management, implants used were K-wires, Schanz pins, and Ellis Plate. The functional outcomes were noted in terms of pain and range of motion, in the follow up of six months. Union was noted clinically and radiologically.

Results: The mean age of the study patients was 42.32 ± 15.77 years. Out of 150 patients, there were 100 (66.67%) male patients. Compared to conservative management, surgical management had significantly lesser time of union (12 vs. 20 weeks, P<0.0001); significantly more excellent results (44% vs. 30%), more good results (32% vs. 15%) (P=0.003); comparable pain score (P=0.236); and comparable functional score (P=0.661). Regarding radiological outcomes, surgical management had significantly more volar tilt (9.6±2.5° vs. 8±5°, P=0.035); lesser Ulnar variance (3±2 vs. 4±2 mm, P=0.004), lesser grip strength <50% (26% vs. 65%, P<0.0001); comparable radial inclination (21±4° vs. 20±5°, P=0.661); and comparable radial height (11±3 vs. 10±5 mm, P=0.195)

Conclusion: To conclude, surgery for distal radius fractures promotes faster healing, lesser pain, lesser malunion, and better functional outcomes. However, it is not without potential risks. Non-surgical treatment is still a suitable option, for patients with contraindications to surgery or having lower need for functional improvement.

## Introduction

Distal radial fractures (DRFs) are commonly seen in orthopedic practice, accounting for over one-sixth of all fractures managed in emergency department. The incidence of DRFs is notably high, ranging from 20 to 40 cases per 10,000 person-years [1,2].

Fractures in adolescents and young adults are often caused by high-energy trauma, such as sports injuries, falls from significant heights, or traffic accidents. On the other hand, elderly individuals are more prone to low-energy fractures due to osteoporosis. DRFs usually result from impact on the outstretched hand. The factors that affect the type of fracture include load’s rate, magnitude and direction [3].

Approximately 90% of radius fractures occur due to stress loading when the wrist is in dorsiflexion. Trauma to the hand can significantly hinder a person's ability to perform daily tasks. Additionally, the management of DRFs has been extensively researched [2].

The management of DRFs is a topic of debate, as various factors like patient characteristics, fracture stability, displacement, and level of activity influence the choice of treatment. Conservative treatment is usually done in case of non-displaced fractures, using either custom orthosis or over-the-counter brace for duration of 4-6 weeks. Displaced fractures, after being reduced, are often treated similarly [2].

The best approach for treating these fractures continues to be a subject of debate, with available options including conservative methods like casting and splinting, as well as different surgical techniques [4]. This wide range of treatments highlights the complexity and variability of the injury, along with factors unique to each patient, such as age, bone health, and functional requirements [5].

The approach to managing DRFs plays a key role in restoration of wrist function and reduction of the risk of complications [6]. For less severe fractures, conservative methods are often preferred, especially in cases where surgery presents more risks. In contrast, surgery is typically recommended for more complicated fractures, particularly in younger, active individuals or when proper anatomical alignment cannot be retained through non-surgical methods [7,8]. Advances in surgical techniques, such as the use of volar locking plates, now offer better options for stable fixation and early rehabilitation. However, these procedures also carry risks, including infections, nerve injuries, and the potential need for hardware removal at a later stage [9].

Not many studies that compared surgical and conservative management specifically for Frykman type III (intra-articular) DRFs have been conducted. Thus, we conducted this study to compare the two techniques in terms of functional outcomes of such fractures which are one of the commonest encountered in our practice.

## Materials and methods

A prospective observational study was done on patients undergoing treatment for closed type of intra-articular DRF Frykman type III. The study was conducted in the “Department of Orthopedics of Government Medical College and Hospital, Jammu” after taking ethical clearance from institution (IEC/GMC/2022/8/8) over a period of 1st November 2020 to 31st October 2021. Frykman Classification were used to classify the fractures [10]. The Frykman classification is a method used to categorize distal radius fractures by how they appear on an X-ray. It evaluates the fracture's pattern, determines whether the radioulnar joint is affected, and checks for any fracture in the distal ulna as well. The system consists of eight types, each with its own subtype [10].

Eligibility criteria was decided to enroll the patients. Inclusion criteria was any patient with radiologically confirmed DRF closed type restricted to radiocarpal joints. Exclusion criteria were patients who did not consent to the study, or were lost to follow up, or suffered from neurological diseases like Parkinsons and Alzheimer’s disease or had open type of DRF.

Sample size

The sample size calculation for this study was based on a previous study by Ochen et al. [6] as per which minimum sample needed for this study was 150, keeping 3.5% margin of error and 5% level of significance.

Procedure

After obtaining written and formal consent for the enrolled patients, demographic details and clinical details were noted, which included age, gender, mode of injury, side of injury, AO classification type and Frykman's classification type. Patients were evaluated after admission and were managed either conservatively or by surgical method. Baseline investigations in terms of hemoglobin, bleeding time (BT), clotting time (CT), blood sugar level, blood urea and creatinine, Urine examination and radiography were done. Radiography included wrist Joint -AP and lateral view, chest X ray PA view, and CT scan of distal radius with wrist. Radiography was done to note the improvement in the injury, where parameters included radial height, radial inclination, volar tilt, ulnar variance and grip strength.

Patients were either managed conservatively (n=100) and surgical management was done in 50 cases. Conservative management was done in the form of cast and medicines. For surgical management, implants used were K-wires of 1 mm, 1.5 mm & 2 mm; Schanz pins of 2.5, 3.5mm; and Ellis Plate. For surgical procedure, the patient was operated under anesthesia.

The outcomes were noted in terms of pain (VAS scores), range of motion, and functional assessment of fingers/ wrist and elbows in the follow up period of 6 months. The functional results were determined using the demerit point system of Saito. This system consists of subjective evaluation, objective evaluation and complications, and the subjective evaluation was graded as excellent, good, and poor [11]. Union was noted clinically and radiologically. Any postoperative complications were noted.

In cases who underwent operative management, a short arm cast was used for stabilization until six weeks with free movement was allowed for the fingers and elbow. Physiotherapy exercises were initiated for the patients for forearm and wrist and under local anesthesia, K-wires were removed after around nine weeks. In the follow-up clinically the patient's functional status, pain, grip strength, and range of motion were assessed

Statistical analysis

**Figure 1:**
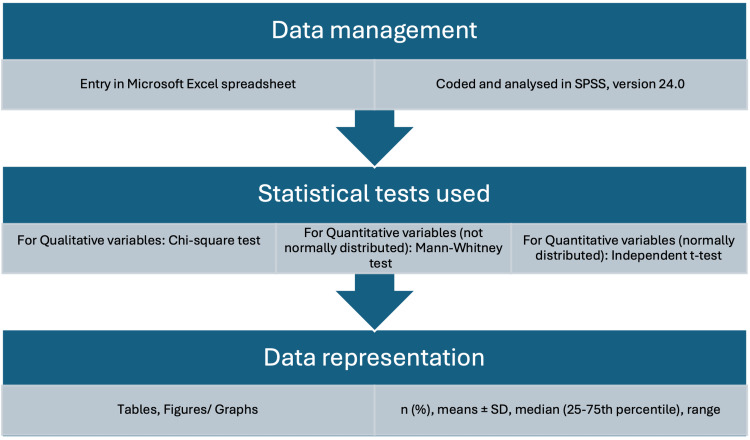
Statistical analysis SPSS:  IBM SPSS Statistics for Windows, Version 24 (Released 2016; IBM Corp., Armonk, New York, United States) Data normality was assessed by the Shapiro-Wilk test. Statistical significance: p< 0.05

Data recording, entry and management was done in a systematic manner as shown in Figure 1.

## Results

The mean age of the patients was 42.32 ± 15.77 years. Out of 150 patients, there were 100 (66.67%) male patients. The most common mode of injury was road traffic accident (RTA) in 66 (44%) patients. Right and left sides were injured in 74 (49.33%) and 76 (50.67%) patients, respectively. As per Frykman classification, all fractures were type III (Table 1).

**Table 1:** Demographic characteristics

Demographic characteristics	n (%)	Mean ± SD
Age (years)	-	42.32 ± 15.77
Gender		
Male	100 (66.67%)	-
Female	50 (33.33%)	
Mode of injury		
Assault	19 (12.67%)	-
Fall from height	21 (14%)	
Road traffic accident	66 (44%)	
Self fall	44 (29.33%)	
Side		
Left	76 (50.67%)	-
Right	74 (49.33%)	

Compared to conservative management, surgical management had significantly lesser time of union (12 vs. 20 weeks, P<0.0001); more excellent results (44% vs. 30%), more good results (32% vs. 15%) (P=0.003); comparable pain score (P=0.236); and comparable functional score (P=0.661) (Table 2).

**Table 2:** Comparison of functional outcome between conservative management and surgical management ^*^Mann-Whitney test, ^†^Chi-square test

Functional outcome	Conservative management (n=100)	Surgical management (n=50)	P-value
Time of union	20 (12 to 16) weeks	12 (12 to 12) weeks	<0.0001^*^
Pain score	26.39-80.77	0-3.09	0.236^*^
Functional score	29 (16 to 79)	32 (24 to 47)	0.661^*^
Results			
Excellent	30 (30%)	22 (44%)	0.003^†^
Good	15 (15%)	16 (32%)	
Fair	35 (35%)	9 (18%)	
Poor	20 (20%)	3 (6%)	

Compared to conservative management, surgical management had significantly more volar tilt (9.6±2.5° vs. 8±5°, P=0.035); lesser Ulnar variance (3±2 vs. 4±2 mm, P=0.004), lesser grip strength <50% (26% vs. 65%, P<0.0001); comparable radial inclination (21±4° vs. 20±5°, P=0.661); and comparable radial height (11±3 vs. 10±5 mm, P=0.195) (Table 3).

**Table 3:** Comparison of radiographic parameters between conservative management and surgical management ^†^Chi-square test, ^‡^Independent t-test

Radiographic parameters	Total	Conservative group	Surgical group	P-value
Radial inclination	20.33±4.7°	20±5°	21±4°	0.661^‡^
Radial height	10.33±4.46 mm	10±5 mm	11±3 mm	0.195^‡^
Volar Tilt	8.53±4.39°	8±5°	9.6±2.5°	0.035^‡^
Ulnar variance	3.67±2.05 mm	4±2 mm	3±2 mm	0.004^‡^
Grip strength<50%	78 (52%)	65 (65%)	13 (26%)	<0.0001^†^

## Discussion

The present study comprehensively assesses the outcomes of conservative or surgical management of intra-articular radius fracture in 150 patients. The mean age of the patients was 42.32 ± 15.77 years.

Among other studies, the mean age of the patients was 40 years in the study by Siddalingamurthy et al. [12], 45.6 years in the study by Awasthi et al. [13], 39 years in the study by Raza et al. [3], and 54 years in the study by Francis et al. [14].

The gender distribution showed that 100 (66.67%) of the patients were male patients and 50 (33.33%) were female patients. Among other studies, male predominance (76.7%) was noted by Siddalingamurthy et al. [12]. There were 67% of men in the study by Raza et al. [3].

In the present study, the commonest mode of injury was RTA (44%). Among other studies, Ravi et al. [15] and Kapoor et al. [16] observed RTA as the commonest mode of injury as seen in 45% and 65% of the patients, respectively. Francis et al. [14] found fall from a height of ≤1 m was seen in 55% of cases (commonest injury mode) [14]. Shahid et al. found that fall from height was the most common mode of injury (62.9%) [17]. Kotian et al. observed that fall from height or on outstretched arm accounted for most of the cases of injury (65%) [18].

In terms of outcomes, the mean time of union was significantly faster in surgical as compared to conservative. This may be because surgery, particularly using modern fixation methods, accelerates bone healing, which is especially advantageous for patients needing a faster return to work or those with higher functional needs. However, it is crucial to balance these advantages against the potential risks of surgery, such as infections or complications from hardware, which were more frequently observed after surgery.

This was similar to that mentioned by Ravi et al. [15] who observed that time for union ≥ months was in more patients after conservative treatment than surgical treatment (55% vs. 12.5%, p=0.002).

Surgical management also allows for lower pain scores as compared to conservative management. The present study found lower pain scores after surgery than conservative management, but the difference was not significant. Our findings are in line with studies that found that surgical management had lesser pain in the follow-up period. Ravi et al. observed that there was a significantly higher mean VAS score after conservative treatment than surgical treatment (20.30±12.19 vs. 16.7±8.97, P=0.07, p=0.07) [15]. This may be because of more stable fracture fixation and the earlier start of rehabilitation after surgical intervention.

In terms of results excellence, excellent results were seen in 30% of the cases in the conservative group as compared to 47.5% in the surgical group. Overall, surgical management was better than conservative management.

The results were also similar to other studies, which also found surgical management to be better than conservative management. Raza et al. [3] observed that excellent results were noted in 80% of cases after surgery and in 70% of cases after conservative management. Ravi et al. [15] found that excellent results were attained in more patients after surgery than conservative treatment (45% vs. 30%).

The selection of either conservative or surgical management is influenced by various factors related to patients, including age, underlying comorbidities, lifestyle, compliance, functional needs, dominant limb, fracture type, severity and alignment of the fracture, soft tissue condition, and additional fractures [15].

Surgical treatment resulted in significantly better radiological outcomes, such as more volar tilt, lesser ulnar variance and grip strength <50%. This was in accordance with the findings of a review article by Song et al. [19], including eight studies, where compared to conservative treatment for distal radius fractures, surgical therapies resulted in significantly better radiographic outcomes (P<0.05) [19]. In another systematic review by Zhu et al. also, better wrist function was observed after surgery as compared to conservative treatment [20].

Limitations of the study

The study holds strength since it was a prospective observational study and follow-up outcomes were assessed for six months of time period. However, as this study was a single-centered study, its results cannot be generalized. Cost-effectiveness of both managements was not evaluated, which constitutes the limitations of the study. The study was restricted to only type III intra-articular DRFs.

## Conclusions

To conclude, surgery for DRFs allows faster healing, lesser pain, lesser malunion, and better functional outcomes. However, non-surgical treatment is still a suitable option for closed intra-articular DRFs, pertinently for patients with contraindications to surgery or having lower need for functional improvement. Overall, treatment decisions should be individually tailored for best outcomes.

## References

[1] Mellstrand-Navarro C, Pettersson HJ, Tornqvist H, Ponzer S (2014). The operative treatment of fractures of the distal radius is increasing: results from a nationwide Swedish study. Bone Joint J.

[2] Patel S, Deshmukh A, Yadav P, Phalak M, Gurnani S, Yadav S, Anand A (2022). Assessment of functional and radiological outcomes of comminuted intra-articular distal radius fracture treated with locking compression plate. Cureus.

[3] Raza A, Saleem HM, Chaudhry M, Khalid MU (2021). Radiological and functional outcome of distal radius fracture treated conservatively vs percutaneous K-wire fixation. Pakistan J Med Health Sci.

[4] Testa G, Vescio A, Di Masi P, Bruno G, Sessa G, Pavone V (2019). Comparison between surgical and conservative treatment for distal radius fractures in patients over 65 years. J Funct Morphol Kinesiol.

[5] Wu M, Li X, Li J, Chen Y (2020). Operative vs conservative treatment in distal radius fractures: a protocol. Medicine (Baltimore).

[6] Ochen Y, Peek J, van der Velde D (2020). Operative vs nonoperative treatment of distal radius fractures in adults: a systematic review and meta-analysis. JAMA Netw Open.

[7] Walenkamp MM, Goslings JC, Beumer A (2014). Surgery versus conservative treatment in patients with type A distal radius fractures, a randomized controlled trial. BMC Musculoskelet Disord.

[8] Ermutlu C, Mert M, Kovalak E, Kanay E, Obut A, Öztürkmen Y (2020). Management of distal radius fractures: comparison of three methods. Cureus.

[9] Alharbi AA, Asiri AM, Al-qahtani AM (2020). Operative vs. conservative management of adult patients with distal radius fractures: a systematic review and meta-analysis. Ann Med Health Sci Res.

[10] Frykman G (1967). Fracture of the distal radius including sequelae--shoulder-hand-finger syndrome, disturbance in the distal radio-ulnar joint and impairment of nerve function. A clinical and experimental study. Acta Orthop Scand.

[11] Fujii K, Henmi T, Kanematsu Y, Mishiro T, Sakai T, Terai T (2002). Fractures of the distal end of radius in elderly patients: a comparative study of anatomical and functional results. J Orthop Surg (Hong Kong).

[12] Siddalingamurthy G, Sheshagiri V, Shreyas MJ (2018). Correlation of functional and radiological outcome of management of distal end radiud fractures using a trinary surgical treatment. Indian J Orthopaed Surg.

[13] Awasthi A, Jadhav S, Taywade S, Salwan A, Khan K (2022). Outcome analysis of distal end radius fractures managed with antegrade intramedullary K-wire fixation. Cureus.

[14] Francis JL, Battle JM, Hardman J, Anakwe RE (2022). Patterns of injury and treatment for distal radius fractures at a major trauma centre. Bone Jt Open.

[15] Ravi KB, Mathew TE, Ganesh T (2019). Conservative versus surgical management of intraarticular fractures of distal end of radius: a comparative clinical study. Int J Orthopaed Sci.

[16] Kapoor H, Agarwal A, Dhaon BK (2000). Displaced intra-articular fractures of distal radius: a comparative evaluation of results following closed reduction, external fixation and open reduction with internal fixation. Int J Care Inj.

[17] Shahid MK, Robati S (2013). The epidemiology and outcome of open distal radial fractures. J Orthop.

[18] Kotian P, Mudiganty S, Annappa R, Austine J (2017). Radiological outcomes of distal radius fractures managed with 2.7mm volar locking plate fixation-a retrospective analysis. J Clin Diagn Res.

[19] Song J, Yu AX, Li ZH (2015). Comparison of conservative and operative treatment for distal radius fracture: a meta-analysis of randomized controlled trials. Int J Clin Exp Med.

[20] Zhu C, Wang X, Liu M, Liu X, Chen J, Liu G, Ji G (2024). Non-surgical vs. surgical treatment of distal radius fractures: a meta-analysis of randomized controlled trials. BMC Surg.

